# From Byproduct to Resource: Fermented Apple Pomace as Beer Flavoring

**DOI:** 10.3390/foods8080309

**Published:** 2019-08-01

**Authors:** Annalisa Ricci, Martina Cirlini, Angela Guido, Claudia Maria Liberatore, Tommaso Ganino, Camilla Lazzi, Benedetta Chiancone

**Affiliations:** Department of Food and Drug, University of Parma, Parco Area delle Scienze 27/A, 43124 Parma, Italy

**Keywords:** apple pomace fermentation, beer flavoring, lactic acid bacteria, volatile profile

## Abstract

One of the main struggles of the large-scale apple processing industry is pomace disposal. One solution for this problem is to convert this waste into a resource. Apple pomace could be used as a substrate for lactic acid bacteria and could induce the formation of a more complex aroma profile, making this fermented product an innovative aromatizer for alcoholic beverages, such as beer. In this study, for the first time, the effect of lacto-fermented apple pomace addition in beer was evaluated. Three bacterial strains (*Lactobacillus rhamnosus* 1473 and 1019, and *L. casei* 2246) were tested for apple pomace fermentation, and *L. rhamnosus* 1473 was the strain that best modified the aromatic profile. The addition of fermented apple pomace to beer increased the complexity of the aroma profile, demonstrating the potential of this byproduct as an aromatizer in the alcoholic beverage industry.

## 1. Introduction

With almost 90 million tons produced all over the world in 2016, apple (*Malus domestica* Borkh.) is one of the most cultivated fruit crops [1], and it can be consumed fresh or as juice, cider, wine, and vermouth, purees, jams, and dried products [2]. 

The apple transformation industry generates large amounts of waste known as ‘apple pomace’ (3600 ktons in 2010), which is mainly composed of skin and flesh (95%), seeds (2% to 4%), and stems (1%) [3]. For the large-scale apple processing industry, pomace disposal is a big problem, impacting massively on the environment; for this reason, in recent years, many efforts have been made to convert this waste into a resource, exploiting its richness in nutrients. Indeed, apple pomace composition varies depending on the variety, origin, and processing technology prior to its generation, but in general, it contains 70% water, 16% carbohydrates, 7% cellulose, 5% protein [4], and many valuable polyphenols with antioxidant properties [5]. Often, only 20% is retrieved as animal feed and the remaining 80% goes to landfill, is incinerated, or is sent to composting sites which results in the release of greenhouse gases [6]. On the contrary, apple pomace waste is an excellent resource for the production of various chemical compounds, such as pectin, limonene, and d-galacturonic acid [7]; moreover, thanks to its abundance of fermentable soluble sugars, including glucose, apple pomace is a perfect substrate for fermentative processes. 

In order to valorize and to find a different use for byproducts obtained by the apple processing industry, the effect of lacto-fermented apple pomace addition to beer has been evaluated for the first time in this study. To reach this main objective, the following aspects were investigated: (i) evaluating the use of three lactic acid bacteria (LAB) strains to ferment apple pomace; (ii) evaluating, per each LAB, the effect on the aromatic profile of apple pomace; (iii) evaluating the effect of a thermal treatment on the volatile profile of unstarted and fermented apple pomace; (iv) evaluating the effect on beer aromatic profile of lacto-fermented apple pomace addition.

## 2. Materials and Methods

### 2.1. Apple Pomace and Bacterial Strain Culture Preparation

Apples, var. Golden Delicious, were centrifuged to extract the juice. The obtained pomace, composed of fruit peel, seeds and apple core, was blended to obtain a more homogeneous substrate for the following fermentations.

*Lactobacillus rhamnosus* 1473 and 1019, and *Lactobacillus casei* 2246, isolated from Parmigiano Reggiano cheese, were singly used as starters for fermentation. All the strains, belonging to the collection of the Food and Drug Department, University of Parma (Italy), were maintained as frozen stocks (−80 °C) in Man Rogosa Sharpe (MRS) medium (Oxoid, Milan, Italy) supplemented with 15% glycerol (*w/v*). The cultures were propagated three times with about 3% (*v/v*) inoculum in MRS and incubated at 37 °C overnight in anaerobic conditions.

### 2.2. Apple Pomace Fermentation

Prior to fermentation, total microbial count was performed on plate count agar (Oxoid, Milan, Italy) at 30 °C for 48 h. In order to remove the endogenous microflora, the apple pomace was sterilized (121 °C for 20 min) and used as substrate for the fermentation process. Each strain was cultivated at 37 °C for 15 h to reach a final concentration of 9 Log CFU/g and then harvested by centrifugation (10,000× *g* for 10 min at 4 °C), washed twice with Ringer’s solution (Oxoid, Milan, Italy), and re-suspended in sterile distilled water. Each culture was inoculated into apple pomace to reach 6 Log CFU/g and incubated at 37 °C for 72 h. Fermentation was developed in sterilized 250 g capacity containers under static conditions.

The growth ability was checked in triplicate at the end of fermentation process by plate count in MRS agar at 37 °C for 48 h under anaerobiosis.

### 2.3. Thermal Treatment on Apple Pomace Volatile Profile

Samples of unstarted and fermented apple pomace were immersed in a water bath at 75 °C for 10 min. After this step, 10 g of each sample was mixed with 90 mL of Tripton soy broth (Oxoid, Milan, Italy) and incubated at 37 °C for 24 h. Next, 0.1 mL of the sample was transferred into 6 mL of medium, incubated at 37 °C for 24 h, and, at the end, the absence of microbial growth was checked by spreading 0.1 mL on MRS agar. In order to evaluate the influence of heat treatment on sterile and *L. rhamnosus* 1473 fermented apple pomace volatile profiles, treated and non-treated samples were analyzed by HS-SPME/GC-MS (Head Space-Solid Phase Microextraction/Gas Chromatography-Mass Spectrometry) technique.

### 2.4. Beer Flavouring

A brewery (Parma, Italy) provided 40 L of its cream ale, which was used as a base to test the apple pomace as beer flavoring. Beer was divided in three 15 L barrels (10 L of beer per barrel) and 2 kg of pomace was added to each, divided as follows: (i) non added beer, (ii) beer with added heated unstarted pomace, (iii) beer with added heated fermented pomace.

After 8 days of “pomace infusion” beers were filtered and bottled; moreover, samples were collected to be analyzed by means of HS-SPME/GC-MS technique. After 30 days of maturation in the bottle, samples were collected from each type of beer and successively analyzed by HS-SPME/GC-MS technique.

### 2.5. Characterization of Volatile Profile of Apple Pomace and Flavored Beer

The volatile profiles of apple pomace samples fermented and unstarted, before and after the heating treatment, and beer samples with and without the addition of apple pomace, at time zero and after the maturation time (30 days), were analyzed by HS-SPME/GC-MS technique, following the protocol reported by Ricci et al. (2018) [8]. Samples of 2 g of apple pomace or 2 mL of beer were used for the analyses, adding 10 µL of an aqueous toluene standard solution (100 µg/mL), useful for the semi-quantification of all detected signals. Head space micro-extraction was performed using a SPME fiber coated with three polymers of different polarity, divinylbenzene–carboxen–polydimethylsiloxane (Supelco, Bellefonte, PA, USA). The analyses were performed on a Thermo Scientific Trace 1300 gas chromatograph coupled to a Thermo Scientific ISQ single quadrupole mass spectrometer equipped with an electronic impact (EI) source (Thermo Fisher Scientific, Waltham, MA, USA), and the separation of analytes was achieved on a SUPELCOWAX 10 capillary column (Supelco, Bellefonte, PA, USA; 30 m × 0.25 mm × 0.25 µm). The characteristic volatiles were identified both by comparing mass spectra with the library (NIST 14) and by calculating their linear retention indices (LRIs).

### 2.6. Statistical Analysis

One-way ANOVA and two-way ANOVA were used to evaluate differences among treatments on apple pomace and on beers; mean separation was performed with Tukey’s test (*p* ≤ 0.05) (SYSTAT 13.1, Systat Software, Inc., Pint Richmond, CA, USA).

In order to verify analogies and differences in beer profiles on the basis of their ingredients and maturation time, the data obtained from volatile profiles determination were statistically elaborated by SPSS Statistics 23.0 software (SPSS Inc., Chicago, IL, USA). In particular, principal component analyses (PCA) were carried out using as variables the concentrations of each volatile detected in the aromatic profiles of the different beer samples. PCA were performed using a covariance matrix and two factors were extracted.

## 3. Results and Discussion

### 3.1. Apple Pomace Fermentation

In order to evaluate the microbial contamination of apple pomace, total microbial count was performed. Since it was 4.14 ± 0.04 Log CFU/g, apple pomace was subjected to sterilization and used assubstrate for the further fermentation process. To this aim, three different strains belonging to *L. rhamnosus* (1473 and 1019) and *L. casei* (2246) species were employed. Results highlighted that all the tested strains were able to grow in apple pomace, increasing their concentrations of 2.52 Log CFU/g for *L. rhamnosus* 1473, 2.66 Log CFU/g for *L. rhamnosus* 1019, and 2.44 Log CFU/g for *L. casei* 2246 in 72 h. The similarities in the growth ability may be due strictly to the genotype–phenotype relationship. Fungi and yeast have been previously used for apple pomace fermentation, producing different compounds such as enzymes [6], citric acid [9], and animal feed [10]. To the best of our knowledge, lactic acid bacteria, mainly employed for lactic acid production [11], have never been used for apple pomace fermentation.

### 3.2. Effect of Different LAB Strains on Volatile Profile of Fermented Apple Pomace

**Figure 1:**
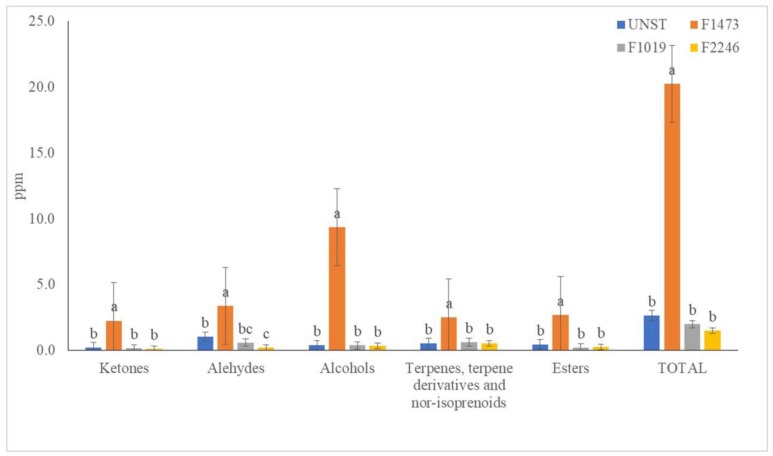
Effect of lactic acid bacteria (LAB) fermentation on volatile composition of apple pomace. One-way analysis of variance (ANOVA), Tukey’s test (*p* ≤ 0.05). letters a–c: mark significant differences/analogies among the samples. UNST: unstarted apple pomace (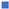); F1473: apple pomace fermented with *Lactobacillus rhamnosus* 1473 (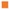); F1019: apple pomace fermented with *Lactobacillus rhamnosus* 1019 (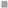); F2246: apple pomace fermented with *Lactobacillus casei* 2246 (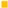).

A total of 45 different compounds were detected in the headspace of all samples and their identification was reported in Table 1. The main classes observed were esters, ketones, terpenes, terpene derivatives, and norisoprenoids and alcohols. The highest significant concentration of total volatile compounds was observed in apple pomace fermented with *L. rhamnosus* 1473 (21.03 ± 1.55 µg/mL) (Figure 1). This strain showed the statistically highest amount of hexanol, previously reported after *L. plantarum* [12] and *L. rhamnosus* [8] fermentation. Among the other detected alcohols, 2-hexenol meaningfully improved its concentration after the employment of 1473 strain. These data, in accordance with an earlier study [8], confirmed that *L. rhamnosus* 1473 is able to improve the quantity of these alcohols in different fruit matrices.

Esters were one of the most representative classes observed in apple pomace fermented with *L. rhamnosus* 1473, increasing their concentration by about seven times compared to the control. Among them, butyl 2-methylbutanoate, hexyl acetate, hexyl n-valerate, and hexyl caproate were the most concentrated compounds. Butyl 2-methylbutanoate a fatty acid ester with a typical apple aroma [26] increased only after *L. rhamnosus* 1473 fermentation and, at the same time, hexyl acetate, found in apple pomace, was preserved only during the fermentation with this strain.. Indeed, in the sample started with *L. rhamnosus* 1473, the resulting amount of hexyl acetate was statistically comparable with the control sample; by contrast, the samples started with *L. casei* 2246 contained a significantly lower concentration of this compound. Hexyl n-valerate and hexyl caproate, typical apple compounds, showed an increase when *L. rhamnosus* 1473 was used. Among aldehydes, benzaldehyde was the most concentrated, especially after *L. rhamnosus* 1473 fermentation, showing a statistically different amount from the other samples (Table S1). Generally, the enzymatic conversion of phenylalanine led to the formation of phenyl pyruvate, and then benzaldehyde was formed by a non-enzymatic step [27].

Concerning terpene compounds, α-farnesene, naturally occurring in apple coating [28], was the sesquiterpene most concentrated (*p* < 0.05) in apple pomace, especially after *L. rhamnosus* 1473 fermentation.

Comparing all the started samples and unfermented apple pomace, *L. rhamnosus* 1473 was the only one showing an increase in total ketone amount, especially sulcatone, which is a flavoring compound found in citrus fruit [29]. Its implementation was observed by Wang, Zhang, Li, & Xu (2013) [30] in grape extract, treated with β-glucosidase, but its increase has never been described after lactic acid fermentation.

Overall, based on these data, *L. rhamnosus* 1473 was the strain that best maintained and/or increased the characteristic aromatic compounds of apple. For this reason, lacto-fermented apple pomace with *L. rhamnosus* 1473 was chosen and used as a beer flavoring agent.

### 3.3. Effect of Heating Treatment on Apple Pomace Volatiles

**Figure 2:**
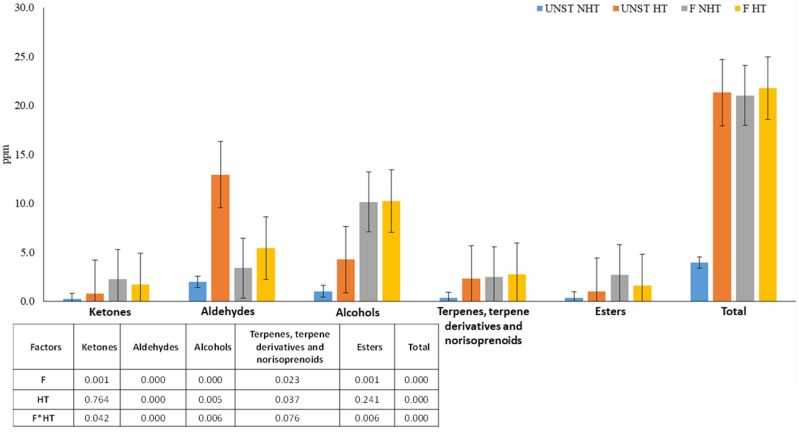
Effect of thermal treatment and of fermentation on volatile composition of apple pomace. Two-way ANOVA, Tukey’s test (*p* ≤ 0.05). UNST NHT: unstarted not heated apple pomace (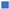); UNST HT: unstarted heated apple pomace (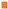); F NHT: *Lactobacillus rhamnosus* 1473 fermented not heated apple pomace (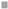); F HT: *Lactobacillus rhamnosus* 1473 fermented heated apple pomace (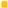).

Both samples of apple pomace, as it is and the fermented one, were submitted to heat treatment before being used as aromatizers for beer. LAB are the predominant beer spoilers and are responsible for 60–90% of beer damage [31]. For this reason, the use of fermented apple pomace as beer flavoring agent will be possible only after a thermal treatment that could eliminate LAB. In order to evaluate the effect of the heating treatment on the volatile profile of the products, all samples were analyzed using the HS-SPME/GC-MS technique. All 45 detected volatile compounds (Table 1) were then quantified (Table S2) and data were statistically elaborated. Results are represented in Figure 2.

Generally, the heating treatment applied to apple pomace led to the formation and/or release of aromatic compounds, especially for the unstarted samples, in which the total volatile amount increased form 3.96 ± 0.14 µg/mL to 21.32 ± 0.45 µg/mL; while for the fermented apple pomace, the heating treatment did not seem to affect the already high total volatile content (21.03 ± 1.55 µg/mL and 21.77 ± 1.62 µg/mL of the unheated and heated samples respectively).

As already observed for apple pomace samples, even if 2-hexenol showed a statistically significant reduction in both unstarted and fermented apple pomaces, hexanol, the main representative volatile of this class, increased after apple pomace fermentation, and was statistically augmented by the thermal treatment.

Esters showed a different trend: statistical analysis of the results demonstrated that their concentration decreased after the thermal treatment in the fermented apple pomace, but they were augmented in the unstarted samples. As previously reported by Kato et al. (2003) [32], the reduction of several esters, such as butyl acetate, hexyl acetate, butyl hexanoate, and hexyl hexanoate, after pasteurization of apple juice can be ascribed to a loss of these volatiles, and not to a chemical modification of the molecules.

Regarding aldehydes, the most relevant increase ascribed to the thermal treatment was noted in the unstarted apple pomace (from 1.99 ± 0.18 µg/mL to 12.93 ± 0.01 µg/mL), while the increase was less considerable in the fermented apple pomace. These results are in accordance with those observed in blackcurrant juice, in which aldehyde and furan quantities were increased after thermal treatments [33]. Among aldehydes, a decrease of hexanal and 2-hexenal in fermented samples could be ascribed to enzymatic activity, as reported in apple already by Su & Wiley (1998) [34]. The concentrations of furfural and benzaldehyde were raised both in unstarted and fermented apple pomace after the thermal treatment because of Maillard reaction, as has been shown for other vegetal matrices as blackcurrant juice [33], but, in our case, the increase remained more restrained in fermented samples. Since furfural and benzaldehyde present bready, caramel, and almond notes, a high content may alter the flavor of apple pomace and generate an intense, cooked sensation. The fermented and heated sample was less abundant in these compounds, and, for this reason, it could be more appreciated as an aromatizer in beer.

The concentration of terpenes increased with the thermal treatment both in unstarted and fermented samples, and was statistically higher in the fermented one. This class of volatile is mainly characterized by the presence of α-farnesene, which increased with the thermal process. This is consistent with the results obtained by Sádecká et al. (2014) [35] in orange juice for other terpenes besides farnesene, such as d-limonene, α-pinene, etc.

Ketone concentration, mainly given by the presence of sulcatone, decreased after the thermal treatment in fermented apple pomace, even though the value resulted higher than the unfermented samples.

The heating step applied to apple pomace samples led to the formation and/or to a release of aromatic compounds in the unstarted product, and did not significantly affect the already high volatile content of the fermented samples. Differently from other studies in which a conventional pasteurization treatment has led to a loss in volatiles, in particular aldehydes such as hexanal and esters such as ethyl acetate and ethyl butyrate [36], the heating process applied in this work permitted enhancement of the flavoring compounds, especially aldehydes, alcohols, and terpenes, so as to maintain the content of aromatic compounds formed during fermentation.

### 3.4. Effect of Apple Pomace Addition on Volatile Profile of Beer

The volatile fractions of beer supplemented with the heated unstarted and fermented apple pomace at time zero and after 30 days of maturation was characterized by HS-SPME/GC-MS technique, and it showed a composition of 59 different aromatic compounds (Table 2; Table S3).

**Figure 3:**
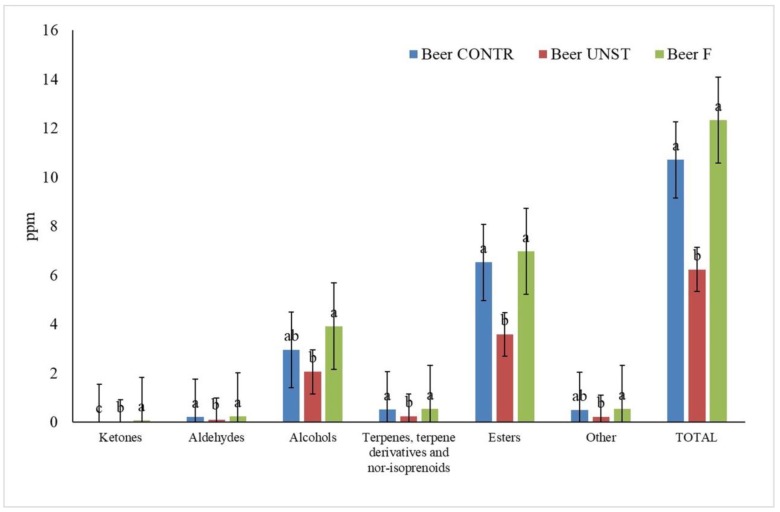
Effect of unstarted and fermented apple pomace addition on volatile composition of beer after 30 days of maturation. One-way ANOVA, Tukey’s test (*p ≤* 0.05). letters a–c: mark significant differences/analogies among the samples. Beer CONTR: Beer with no added apple pomace (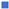); Beer UNST: beer with added unstarted apple pomace (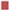); Beer F: beer with added *Lactobacillus rhamnosus* 1473 fermented heated apple pomace (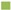).

As observed for apple pomace, the compounds observed in beer also belonged to five chemical classes: esters, aldehydes, ketones, alcohols, and terpenes. The most abundant class, both for number and quantity of molecules, was that of esters (Figure 3). Our results showed that the total amount of esters was statistically comparable between the control sample and the beer aromatized with fermented apple pomace, while the beer with added unstarted apple pomace showed a lower quantity of esters. Within this class, the main representative compounds were ethyl acetate, present also in apple pomace, and ethyl octanoate, characteristic of beer volatile profile [37].

The second main class of aromatic compounds represented was that of alcohols, mostly represented by isoamyl alcohol, phenylethyl alcohol, hexanol, and isobutyl alcohol, as demonstrated in previous studies [37]. The total alcohol amount showed the same trend of esters. Some alcohols were detected in higher amounts in aromatized beer, because they were characteristic of apple: 2-ethy-1-hexanol, benzyl alcohol, hexanol, isobutyl alcohol, and phenylethyl alcohol. Moreover, these compounds were statistically more concentrated in beer flavored with fermented apple pomace than in control or in beer flavored with apple pomace.

A similar trend was also observed for terpenes and derivatives and for aldehydes. Even if the total amount of terpenes, terpene derivatives, and aldehydes was similar between control samples and beer in which fermented apple pomace was used as an aromatizer, the concentration of α-farnesene and nonanal was more elevated in this last sample, demonstrating again the contribution of fermented apple pomace.

Ketones showed a higher concentration in beer supplemented with fermented apple pomace, and the presence of sulcatone, with characteristic citrus notes, could be ascribed to the addition of apple pomace.

Since commercial beers are subjected to a maturation time of 30 days before their commercialization, the beer samples considered in this study were analyzed at time zero and 30 days after bottling, to follow the evolution of the volatile fraction. The profiles obtained after the maturation time were comparable with those of the starting samples, in terms of volatile composition, while quantitatively, a reduction of the total aromatic compound amount was observed in all the beer samples after 30 days.

**Figure 4:**
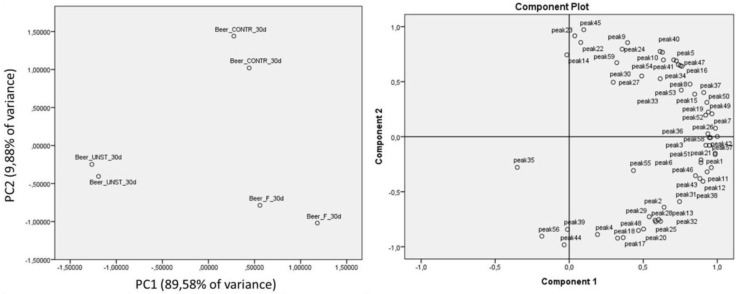
Principal component analysis (PCA) of beer and flavored beer. Scatter plot of scores from PC1 versus PC2 obtained using the concentrations (ppm) of the 59 different detected volatile compounds observed for beer samples analyzed after 30 days of maturation, and the relative loadings of the variables used. Beer CONTR: Beer with no added apple pomace; Beer UNST: beer with added unstarted apple pomace; Beer F: beer with added *Lactobacillus rhamnosus* 1473 fermented heated apple pomace; analyzed at 30 days of maturation.

In order to underline differences and/or analogies among beer samples on the basis of the ingredient used for the aromatization after the maturation time, principal component analysis (PCA) was performed, using as independent variables the concentrations of all the detected volatile compounds (59 variables corresponding to the 59 identified compounds, listed in Table 2), extracting two components and applying a covariance matrix. The first two components explained more than the 99% of the total variance, as shown in Figure 4. PC1 was important in discrimination between beer with added heated apple pomace (Beer_UNST) and beer with added heated fermented apple pomace (Beer_F). This differentiation was possible thanks to benzaldehyde (peak 35) amount, higher in samples with the added sterilized product, and to 2-nonanone (peak 20), hexyl n-valerate (peak 25) and 2-ethyl-1-hexanol (peak 32) concentrations, more elevated in samples aromatized with fermented apple pomace. Component 2 contributed to distinguishing aromatized beers form their relative control samples (Beer_CONTR). After 30 days of maturation, control samples were indeed poor in 2-heptenal (peak 14), 2-hexenol (peak 22), and butyl caproate (peak 23), volatiles characteristic of apple pomace, but at the same time, methyl geranate (peak 45) was better maintained in these samples with respect to aromatized beers.

It is possible to conclude that the statistical unsupervised approach applied to data, PCA, represented a useful tool to determine the contributions of the addition of heated apple pomace, fermented and unstarted, to beer.

## 4. Conclusions

This study investigated, for the first time, the use of lacto-fermented apple pomace as an aromatizer for beer. Results demonstrated that lactic acid bacteria can grow and modify the volatile profile of this by-product, leading to a beer characterized by distinctive aromatic features. Future studies will be focused on the exploitation of different apple varieties as substrates for fermentation. At the same time, a large number of lactic acid bacteria will be used as a starter for apple pomace fermentation in order to increase the knowledge on fermented apple pomace as a beer flavoring and to produce artisanal fruit beers which could satisfy consumer expectations.

## Figures and Tables

**Table 1:** Volatile compounds characteristic of apple pomace. For each volatile compound aromatic note, calculated and tabulated linear retention indices (LRIs) and references are reported.

Peak Number	Identification	Aromatic Note	LRI Calc.	LRI Litt.	Reference
1	Hexanal	Herbal	1078	1085	[13]
2	n.i.		1079		
3	Isoamyl acetate	Fruity, banana	1113	1121	[14]
4	Butanol	Fruity, wine	1141	1166	[15]
5	2-Heptanone	Cheesy	1184	1188	[15]
6	Heptanal	Herbal	1186	1191	[16]
7	d-Limonene	Citrus	1196	1194	[15]
8	n.i.		1214		
9	2-Hexenal	Apple, green	1220	1208	[13]
10	Isoamyl alcohol	Alcoholic, whiskey	1221	1217	[15]
11	Butyl 2-methylbutanoate	Fruity, green	1225		
12	Pentanol	Fermented	1247	1251	[8]
13	Ethyl isoamyl ketone		1253		
14	Hexyl acetate	Fruit, herb	1270	1290	[15]
15	Octanal	Aldehydic	1287	1297	[16]
16	2-Heptenal	Green	1322	1333	[17]
17	Sulcatone	Citrus	1335	1338	[13]
18	Hexanol	Herbal	1349	1344	[18]
19	Nonanal	Aldehydic	1390	1396	[14]
20	2-Hexenol	Leaf, green	1402	1410	[19]
21	Butyl caproate	Fruity, pineapple, apple	1407	1407	[13]
22	Hexyl butyrate	Green	1411	1406	[18]
23	Hexyl n-valerate	Fruity	1422	1487	[20]
24	1-Octen-3-ol	Earthy	1445	1455	[17]
25	Isoamyl caproate	Fruity	1454	1464	[21]
26	Furfural	Bready, caramel	1467	1475	[16]
27	2-Ethyl-1-hexanol	Citrus	1483	1500	[15]
28	Benzaldehyde	Fruity, almond	1524	1526	[15]
29	Linalool	Floral	1543	1547	[8]
30	Octanol	Waxy	1553	1567	[15]
31	Hexyl caproate	Green	1606	1606	[18]
32	Capric acid, ethyl ester	Waxy	1633	1654	[16]
33	2-Decenal	Waxy	1641	1627	[22]
34	Estragole	Licorice, anise	1668	1655	[23]
35	Ethyl 9-decenoate	Fruity	1685	1697	[24]
36	α-Farnesene (Z, E)	Sweet, wood	1717	1727	[24]
37	α-Farnesene (E, E)	Sweet, wood	1744	1740	[25]
38	Hexyl caprylate	Green	1803	1804	[21]
39	2,4-Decadienal/2-phenylethyl acetate	Seaweed/floral	1807		
40	β-Damascenone	Woody, sweet, fruity	1817	1820	[8]
41	Trans-Geraniol	Sweet, floral, fruity	1840	1845	[8]
42	Geranyl acetone	Magnolia, green	1849	1867	[21]
43	Benzyl alcohol	Sweet, floral	1875	1876	[15]
44	Phenylethyl alcohol	Floral	1909	1931	[14]
45	Eugenol	Spicy	2151	2157	[8]

LRI Calc. Linear Retention Indices calculated; LRI Litt. Linear Retention Indices tabulated; n.i. not identified.

**Table 2:** Volatile compounds characteristic of beer samples. For each volatile compound aromatic note, calculated and tabulated LRIs and references are reported.

Peak Number	Identification	Aromatic Note	LRI Calc.	LRI Litt.	Reference
1	Ethyl acetate	Ethereal, fruity	850	855	[8]
2	Ethyl butyrate	Apple	1032	1037	[16]
3	Butyl acetate	Pear	1068	1075	[13]
4	Hexanal	Herbal	1078	1085	[13]
5	Isobutyl isobutyrate	Fruity	1082	1092	[38]
6	Isobutyl alcohol	Ethereal	1100	1108	[14]
7	Isoamyl acetate	Fruity, banana	1115	1121	[14]
8	Myrcene	Peppery, terpenic	1149	1164	[16]
9	Isoamyl propionate	Fruity	1183	1192	[39]
10	2-Methylbutyl isobutyrate	Fruity	1190	1199	[21]
11	Isoamyl alcohol	Alcoholic, whiskey	1210	1217	[15]
12	Ethyl caproate	Fruity	1231	1240	[15]
13	Hexyl acetate	Fruit, herb	1270	1281	[15]
14	2-Heptenal	Green	1322	1333	[17]
15	Ethyl heptanoate	Fruity, pineapple	1328	1327	[21]
16	Non-identified compound deriving from hop used as bittering agent	Herbal	1330		
17	Sulcatone	Citrus	1335	1338	[13]
18	Hexanol	Herbal	1349	1344	[18]
19	3-Hexenol	Green, grassy, melon	1381	1387	[40]
20	2-Nonanone	Fruity	1387	1395	[17]
21	Nonanal	Aldehydic	1390	1396	[14]
22	2-Hexenol	Leaf, green	1402	1410	[19]
23	Butyl caproate	Fruity, pineapple, apple	1407	1407	[13]
24	Hexyl butyrate	Green	1411	1406	[18]
25	Hexyl n-valerate	Fruity	1422	1498	[21]
26	Ethyl octanoate	Waxy	1433	1443	[16]
27	1-Octen-3-ol	Earthy	1445	1455	[17]
28	Heptanol	Mushroom	1451	1460	[14]
29	Isoamyl caprote	Fruity	1454	1464	[21]
30	Sulcatol	Green	1459	1461	[21]
31	Furfural	Bready, caramel	1467	1475	[16]
32	2-Ethyl-1-hexanol	Citrus	1483	1475	[16]
33	Decanal	Orange peel	1492	1507	[16]
34	2-Nonanol	Fresh, cucumber	1514	1528	[21]
35	Benzaldehyde	Fruity, almond	1524	1526	[15]
36	Ethyl nonanoate	Waxy	1531	1528	[39]
37	Linalool	Floral	1543	1547	[8]
38	Octanol	Waxy	1553	1567	[15]
39	Hexyl caproate	Green	1606	1606	[18]
40	2-Decanol		1613	1621	[21]
41	Capric acid, ethyl ester	Waxy	1633	1654	[16]
42	Phenyl acetaldehyde	Green, honey	1642	1671	[41]
43	α-Caryophyllene	Spicy	1660	1680	[42]
44	Ethyl-9-decenoate	Fruity	1685	1697	[24]
45	Methyl geranate	Floral, green, fruity	1690	1686	[21]
46	Dihydroterpineol	Pine	1692		
47	2-Undecanol	Waxy	1712	1706	[21]
48	α-Farnesene (E, E)	Sweet, wood	1744	1740	[25]
49	Decanol	Fatty	1755	1774	[16]
50	Citronellol	Floral, rose	1760	1779	[16]
51	Cis-geraniol	Sweet, floral, fruity, rose	1794	1788	[8]
52	2,4-Decadienal/2-phenylethyl acetate	Seaweed/floral	1807		
53	Ethyl laurate	Waxy	1835	1867	[16]
54	trans-Geraniol	Sweet, floral, fruity	1840	1845	[8]
55	Geranyl acetone	Magnolia, green	1849	1867	[21]
56	Benzyl alcohol	Sweet, floral	1875	1876	[15]
57	Phenylethyl alcohol	Floral	1909	1931	[14]
58	Caprylic acid	Fatty	2074	2098	[41]
59	Eugenol	Spicy	2151	2157	[8]

## Supplementary Materials

The following are available online at https://www.mdpi.com/2304-8158/8/8/309/s1, Table S1: Influence of LAB strain fermentation on the volatile profile of apple pomace, Table S2: Influence of heating treatment and fermentation with *Lactobacillus rhamnosus* 1473 on the volatile profile of apple pomace, Table S3: Influence of time of maturation and of the addition of apple pomace, fermented with *Lactobacillus rhamnosus* 1473, on the volatile profile of beer.
