# Supplementary material for: From Byproduct to Resource: Fermented Apple Pomace as Beer Flavoring
Source: Foods. 2019 Aug 1;8(8):309. doi: 10.3390/foods8080309 (PMC6723389; doi:10.3390/foods8080309)
Supplement: Supplementary file 1 [file foods-08-00309-s001.pdf]

Supplementary material

Table S1. Influence of LAB strain fermentation on the volatile profile of apple pomace.

**Compounds**	**Unstarted**			**F1473**			**F1019**			**F2246**		
	**ppm**	**±SD**		**ppm**	**±SD**		**ppm**	**±SD**		**ppm**	**±SD**	
**Ketones**	0.226	0.05	b	2.258	0.41	a	0.162	0.03	b	0.138	0.02	b
2-Heptanone	0.001	0.00	b	0.051	0.00	a	0.002	0.00	b	0.003	0.00	b
Ethyl isoamyl ketone	0.000	0.00	b	0.200	0.00	a	0.000	0.00	b	0.000	0.00	b
Sulcatone	0.226	0.05	b	2.188	0.41	a	0.160	0.03	b	0.136	0.02	b
**Alehydes**	1.030	0.23	b	3.396	0.21	a	0.597	0.06	b	0.207	0.02	b
2,4-decadienal/2-Phenylethylacetate	0.002	0.00		0.017	0.01		0.000	0.00		0.001	0.00	
2-Decenal	0.006	0.00	a	0.001	0.00	b	0.000	0.00	b	0.000	0.00	b
2-Heptenal	0.032	0.01	a	0.014	0.00	ab	0.018	0.01	ab	0.002	0.00	b
2-Hexenal	0.235	0.07	a	0.058	0.00	b	0.083	0.01	b	0.000	0.00	b
Benzaldehyde	0.609	0.15	b	3.278	0.19	a	0.407	0.08	b	0.132	0.00	
Furfural	0.016	0.00	b	0.025	0.00	b	0.085	0.01	a	0.035	0.01	
Hexanal	0.081	0.01	a	0.001	0.00	bc	0.001	0.00	c	0.034	0.01	b
Heptanal	0.005	0.00		0.000	0.00		0.000	0.00		0.000	0.00	
Nonanal	0.037	0.01	a	0.003	0.00	b	0.000	0.00	b	0.000	0.00	b
Octanal	0.007	0.00	a	0.002	0.00	b	0.002	0.00	b	0.002	0.00	b
**Alcohols**	0.393	0.01	b	9.363	0.32	a	0.389	0.01	b	0.362	0.01	b
1-Octen-3-ol	0.005	0.01	b	0.087	0.00	a	0.015	0.00	b	0.016	0.00	b
2-ethyl-1-hexanol	0.004	0.00		0.017	0.01		0.000	0.00		0.000	0.00	
2-Hexenol	0.025	0.01	b	0.802	0.02	a	0.000	0.00	b	0.000	0.00	b
Benzyl alcohol	0.005	0.00	b	0.343	0.03	a	0.028	0.00	b	0.045	0.00	b
Butanol	0.008	0.00	b	0.052	0.00	a	0.016	0.00	b	0.013	0.00	b
Hexanol	0.318	0.01	b	7.906	0.31	a	0.307	0.00	b	0.246	0.01	b
Isoamyl alcohol	0.000	0.00		0.000	0.00		0.000	0.00		0.000	0.00	
Octanol	0.008	0.00	b	0.074	0.00	a	0.014	0.00	b	0.011	0.00	b
Pentanol	0.007	0.00	b	0.081	0.00	a	0.008	0.00	b	0.015	0.00	b
Phenylethyl alcohol	0.013	0.00	a	0.001	0.00	b	0.001	0.00	b	0.015	0.01	a
**Terpenes, terpene derivatives and nor-isoprenoids**	0.540	0.11	b	2.506	0.08	a	0.632	0.12	b	0.521	0.12	b
D-Limonene	0.002	0.00	b	0.013	0.00	a	0.001	0.00	b	0.000	0.00	b
cis-Geraniol	0.005	0.00	b	0.000	0.00	c	0.006	0.00	a	0.004	0.00	b
Estragole	0.005	0.00		0.008	0.00		0.005	0.00		0.005	0.00	
Eugenol	0.002	0.10	b	0.013	0.07	a	0.004	0.10	b	0.002	0.11	b

Geranyl acetone	0.000	0.00	b	0.000	0.01	b	0.003	0.01	a	0.003	0.01	a
Linalool	0.008	0.00	b	0.030	0.00	a	0.011	0.00	b	0.011	0.00	b
α-Farnesene (E, E)	0.498	0.00	b	2.323	0.00	a	0.553	0.00	b	0.455	0.00	b
α-Farnesene (Z, E)	0.012	0.00	b	0.093	0.00	a	0.049	0.00	b	0.034	0.00	b
β-Damascenone	0.009	0.00	b	0.026	0.00	a	0.000	0.00	c	0.008	0.00	b
**Esters**	0.455	0.02	b	2.712	0.47	a	0.210	0.03	b	0.251	0.07	b
Butyl 2-methylbutanoate	0.011	0.00	b	0.183	0.04	a	0.000	0.00	b	0.000	0.00	b
Butyl caproate	0.038	0.01	b	0.200	0.07	a	0.049	0.01	b	0.055	0.06	b
Capric acid, ethyl ester	0.000	0.00		0.000	0.00		0.000	0.00		0.000	0.00	
Ethyl-9-decenoate	0.000	0.00		0.000	0.00		0.000	0.00		0.000	0.00	
Hexyl acetate	0.122	0.01	a	0.162	0.04	a	0.000	0.00	b	0.000	0.00	b
Hexyl butyrate	0.026	0.00	b	0.001	0.00	b	0.123	0.03	a	0.050	0.01	b
Hexyl caproate	0.135	0.02	b	0.634	0.03	a	0.037	0.00	c	0.147	0.00	b
Hexyl n-valerate	0.117	0.00	b	1.498	0.46	a	0.000	0.00	c	0.000	0.00	c
Isoamyl acetate	0.000	0.00		0.000	0.00		0.000	0.00		0.000	0.00	
Isoamyl caproate	0.007	0.00	b	0.034	0.00	a	0.000	0.00	b	0.000	0.00	b
**TOTAL**	2.654	0.42	b	20.235	1.48	a	1.998	0.12	b	1.483	0.08	b

One-way analysis of variance (ANOVA), followed by Tukey's test; $p \leq 0.05$. Per each row, values followed by different letters are statistically different.

Table S2. **INFLUENCE OF HEATING TREATMENT AND FERMENTATION WITH *LACTOBACILLUS RHAMNOSUS* 1473 ON THE VOLATILE PROFILE OF APPLE POMACE.**

**Compounds**	**Unstarted**				**F1473**				**Statistical analysis**		
									**F**	**HT**	**F*HT**
	**NHT (ppm)**	**±SD**	**HT (ppm)**	**±SD**	**NHT (ppm)**	**±SD**	**HT (ppm)**	**±SD**		**p**	
**Ketones**	0.226	**0.05**	0.792	0.02	2.258	0.41	1.743	0.27	0.001	0.892	0.037
2-Heptanone	0.000	0.00	0.000	0.00	0.051	0.00	0.053	0.00	0.000	0.563	0.581
Ethyl isoamyl ketone	0.000	0.00	0.000	0.00	0.020	0.00	0.004	0.00	0.000	0.001	0.001
Sulcatone	0.226	0.05	0.792	0.02	2.188	0.41	1.686	0.27	0.001	0.864	0.038
**Aldehydes**	1.994	0.18	12.929	0.01	3.396	0.21	5.412	0.18	0.000	0.000	0.000
Hexanal	0.339	0.06	1.778	0.33	0.001	0.00	0.001	0.00	0.001	0.004	0.004
2,4- Decadienal/2-phenylethyl acetate	0.005	0.00	0.038	0.00	0.017	0.00	0.005	0.01	0.000	0.008	0.051
2-Decenal	0.006	0.00	0.016	0.01	0.001	0.01	0.009	0.00	0.008	0.002	0.508

2-Heptenal	0.011	0.00	0.032	0.00	0.014	0.00	0.067	0.01	0.001	0.000	0.001
2-Hexenal	0.235	0.07	1.236	0.23	0.058	0.00	0.166	0.04	0.002	0.003	0.007
Benzaldehyde	1.337	0.00	9.602	0.00	3.278	0.00	5.102	0.00	0.000	0.000	0.000
Furfural	0.016	0.05	0.050	0.10	0.025	0.19	0.029	0.23	0.125	0.005	0.012
Heptanal	0.005	0.00	0.035	0.00	0.000	0.00	0.002	0.00	0.000	0.000	0.000
Nonanal	0.035	0.00	0.119	0.01	0.003	0.00	0.028	0.01	0.001	0.001	0.009
Octanal	0.005	0.00	0.023	0.00	0.000	0.00	0.005	0.00	0.001	0.001	0.007
**Alcohols**	0.986	0.07	3.990	0.29	9.363	0.32	9.676	0.56	0.000	0.005	0.006
1-Octen-3-ol	0.003	0.00	0.029	0.01	0.087	0.00	0.045	0.00	0.000	0.022	0.000
2-Ethyl-1-hexanol	0.004	0.00	0.023	0.01	0.017	0.01	0.025	0.01	0.142	0.032	0.278
2-Hexenol	0.090	0.00	0.021	0.00	0.802	0.02	0.610	0.01	0.000	0.000	0.002
Benzyl alcohol	0.007	0.00	0.013	0.00	0.343	0.03	0.030	0.00	0.000	0.000	0.000
Butanol	0.004	0.00	0.011	0.00	0.052	0.00	0.040	0.00	0.000	0.168	0.005
Hexanol	0.862	0.06	3.828	0.27	7.906	0.31	8.186	0.58	0.000	0.003	0.006
Isoamyl alcohol	0.000	0.00	0.000	0.00	0.000	0.00	0.000	0.00	0.000	0.000	0.001
Octanol	0.009	0.00	0.039	0.00	0.074	0.00	0.069	0.00	0.000	0.001	0.000
Pentanol	0.006	0.00	0.023	0.00	0.081	0.00	0.049	0.00	0.000	0.018	0.000
Phenylethyl alcohol	0.001	0.00	0.004	0.00	0.001	0.00	0.621	0.02	0.000	0.000	0.000
**Terpenes, terpene derivatives and nor-isoprenoids**	0.194	0.30	0.020	0.01	2.506	0.08	2.756	1.02	0.022	0.037	0.075
D-Limonene	0.002	0.00	0.000	0.00	0.013	0.00	0.017	0.00	0.001	0.545	0.114
Estragole	0.003	0.00	0.009	0.00	0.008	0.00	0.010	0.00	0.073	0.014	0.123
Eugenol	0.001	0.00	0.003	0.00	0.013	0.00	0.018	0.00	0.000	0.025	0.243
Geraniol	0.000	0.00	0.000	0.00	0.000	0.00	0.000	0.00	0.526	0.286	0.311
Geranyl acetone	0.000	0.00	0.000	0.00	0.000	0.00	0.000	0.00	0.001	0.002	0.019
Linalool	0.003	0.00	0.015	0.00	0.030	0.00	0.030	0.00	0.001	0.048	0.037
α-farnesene	0.315	0.00	2.206	0.02	2.323	0.07	2.588	1.01	0.029	0.039	0.086
α-farnesene (Z,E)	0.012	0.00	0.071	0.00	0.093	0.01	0.077	0.00	0.000	0.001	0.000
β-Damascenone	0.001	0.00	0.008	0.00	0.026	0.00	0.017	0.00	0.000	0.326	0.002
**Esters**	0.370	0.02	1.019	0.07	2.712	0.47	1.600	0.04	0.000	0.188	0.003
Butyl 2-methylbutanoate	0.011	0.00	0.000	0.00	0.183	0.04	0.092	0.01	0.001	0.017	0.037
Butyl caproate	0.025	0.00	0.072	0.00	0.200	0.07	0.098	0.00	0.013	0.311	0.034
Capric acid, ethyl ester	0.000	0.00	0.000	0.00	0.000	0.00	0.000	0.00	0.000	0.000	0.001
Ethyl 9-decenoate	0.000	0.00	0.000	0.00	0.000	0.00	0.000	0.00	0.001	0.004	0.010
Hexyl acetate	0.122	0.01	0.138	0.02	0.162	0.04	0.168	0.03	0.161	0.611	0.812
Hexyl butyrate	0.000	0.00	0.000	0.00	0.001	0.00	0.001	0.00	0.000	0.422	0.002
Hexyl caproate	0.088	0.01	0.240	0.07	0.634	0.03	0.494	0.00	0.000	0.826	0.004
Hexyl n-valerate	0.117	0.00	0.523	0.02	1.498	0.46	0.721	0.00	0.008	0.314	0.021
Isoamyl acetate	0.000	0.00	0.000	0.00	0.000	0.00	0.000	0.00	0.000	0.000	0.000

Isoamyl caproate	0.007	0.00	0.046	0.00	0.034	0.00	0.025	0.00	0.085	0.000	0.000
**TOTAL**	4.079	0.14	21.649	0.45	22.005	1.55	22.419	1.62	0.000	0.000	0.000

Two-way analysis of variance (ANOVA) ($p \leq 0.05$). NHT: not heated apple pomace. HT: heated apple pomace. F: fermentation with *Lactobacillus rhamnosus* 1473.

Table S3: Influence of time of maturation and of the addition of apple pomace, fermented with *Lactobacillus rhamnosus* 1473, on the volatile profile of beer.

Compounds	T0						T30						Statistical analysis		
	Beer CONTR (ppm)	±SD	Beer UNST (ppm)	±SD	Beer F (ppm)	±SD	Beer CONTR (ppm)	±SD	Beer UNST (ppm)	±SD	Beer F (ppm)	±SD	T	F	T*F
													p		
**Ketones**	0,247	0,06	0,273	0,06	0,377	0,07	0,003	0,00	0,030	0,00	0,074	0,01	0,000	0,047	0,589
2-Nonanone	0,000	0,00	0,009	0,00	0,032	0,00	0,001	0,00	0,004	0,00	0,011	0,00	0,001	0,000	0,001
Sulcatone	0,247	0,06	0,264	0,06	0,344	0,07	0,002	0,00	0,026	0,00	0,063	0,01	0,000	0,095	0,754
**Aldehydes**	0,993	0,15	1,076	0,20	1,127	0,21	0,216	0,01	0,100	0,01	0,248	0,02	0,000	0,561	0,600
2,4-decadienal/2-phenylethyl acetate	0,926	0,12	0,787	0,16	0,787	0,14	0,093	0,00	0,054	0,00	0,095	0,01	0,000	0,459	0,615
2-Heptenal	0,000	0,00	0,014	0,00	0,015	0,00	0,001	0,00	0,000	0,00	0,000	0,00	0,000	0,004	0,002
Benzaldehyde	0,000	0,00	0,113	0,03	0,046	0,01	0,004	0,00	0,005	0,00	0,004	0,00	0,001	0,002	0,002
Decanal	0,000	0,00	0,041	0,00	0,056	0,02	0,021	0,01	0,005	0,00	0,015	0,00	0,014	0,026	0,007
Furfural	0,000	0,00	0,024	0,00	0,089	0,01	0,003	0,00	0,002	0,00	0,013	0,01	0,000	0,000	0,000
Hexanal	0,000	0,00	0,000	0,00	0,004	0,00	0,001	0,00	0,002	0,00	0,003	0,00	0,831	0,002	0,052
Nonanal	0,066	0,02	0,049	0,01	0,078	0,02	0,083	0,02	0,029	0,01	0,105	0,00	0,446	0,014	0,220
Phenylacetaldehyde	0,000	0,00	0,049	0,00	0,052	0,01	0,011	0,00	0,003	0,00	0,014	0,00	0,000	0,000	0,000

**Alcohols**	24,940	1,34	25,371	5,24	25,811	3,55	2,957	0,14	2,056	0,11	3,921	0,39	0,000	0,815	0,914
1-Octen-3-ol	0,037	0,00	0,038	0,01	0,059	0,02	0,006	0,00	0,004	0,00	0,005	0,00	0,021	0,033	0,019
2-Ethyl-1-hexanol	0,000	0,00	0,047	0,01	0,320	0,07	0,000	0,00	0,006	0,00	0,056	0,01	0,000	0,009	0,039
2-Decanol	0,226	0,01	0,104	0,01	0,093	0,01	0,032	0,00	0,010	0,00	0,019	0,00	0,000	0,000	0,000
2-Hexenol	0,000	0,00	0,001	0,00	0,006	0,00	0,001	0,00	0,000	0,00	0,000	0,00	0,021	0,033	0,019
2-Nonanol	0,456	0,01	0,320	0,05	0,297	0,05	0,064	0,00	0,026	0,00	0,047	0,01	0,000	0,914	0,890
2-Undecanol	0,136	0,03	0,084	0,01	0,114	0,01	0,041	0,00	0,010	0,00	0,028	0,00	0,000	0,016	0,579
3-Hexenol	0,001	0,00	0,000	0,00	0,000	0,00	0,053	0,01	0,024	0,00	0,056	0,01	0,000	0,013	0,013
Benzyl alcohol	0,000	0,00	0,116	0,03	0,151	0,02	0,003	0,00	0,024	0,01	0,025	0,01	0,000	0,000	0,002
Decanol	0,056	0,02	0,061	0,01	0,066	0,01	0,016	0,00	0,009	0,00	0,016	0,00	0,000	0,538	0,566
Hexanol	0,000	0,00	0,949	0,28	1,329	0,22	0,007	0,01	0,085	0,00	0,191	0,02	0,000	0,001	0,004
Heptanol	0,063	0,01	0,013	0,00	0,117	0,03	0,009	0,00	0,010	0,00	0,017	0,00	0,001	0,004	0,007
Isoamyl alcohol	11,848	0,16	12,643	2,32	11,064	0,86	1,526	0,10	1,039	0,04	2,012	0,21	0,000	0,916	0,282
Isobutyl alcohol	0,379	0,06	0,567	0,02	0,477	0,16	0,095	0,01	0,052	0,03	0,133	0,00	0,000	0,350	0,143
Octanol	0,255	0,03	0,252	0,05	0,251	0,04	0,035	0,00	0,026	0,00	0,045	0,01	0,000	0,914	0,890
Phenylethyl alcohol	11,484	1,37	10,156	2,41	11,452	2,06	1,068	0,07	0,730	0,04	1,270	0,12	0,000	0,621	0,877
Sulcatol	0,000	0,00	0,019	0,00	0,014	0,00	0,002	0,00	0,000	0,00	0,001	0,00	0,000	0,002	0,001
**Terpenes, terpene derivatives and nor-isoprenoids**	3,946	0,35	3,173	0,64	3,516	0,47	0,523	0,02	0,252	0,02	0,553	0,02	0,000	0,195	0,575
α-Caryophyllene	0,193	0,03	0,146	0,02	0,506	0,03	0,058	0,00	0,024	0,00	0,112	0,00	0,000	0,000	0,000

α-Farnesene (E, E)	0,061	0,01	0,426	0,09	0,594	0,06	0,004	0,00	0,022	0,01	0,078	0,00	0,000	0,000	0,001
Cis-geraniol	0,000	0,00	0,000	0,00	0,021	0,00	0,003	0,00	0,002	0,00	0,004	0,00	0,000	0,000	0,000
Citronellol	0,076	0,01	0,139	0,02	0,129	0,01	0,025	0,00	0,010	0,00	0,024	0,00	0,000	0,026	0,006
Dihydroterpineol	0,000	0,00	0,110	0,02	0,066	0,02	0,002	0,00	0,001	0,00	0,004	0,00	0,000	0,000	0,000
Eugenol	0,000	0,00	0,000	0,00	0,000	0,00	0,009	0,01	0,002	0,00	0,004	0,00	0,038	0,358	0,349
Geranyl acetone	0,000	0,00	0,018	0,00	0,019	0,00	0,004	0,01	0,004	0,00	0,008	0,00	0,006	0,004	0,011
Linalool	2,660	0,32	2,011	0,44	1,872	0,36	0,292	0,01	0,160	0,01	0,265	0,02	0,000	0,123	0,197
Methyl geranate	0,093	0,01	0,041	0,00	0,043	0,01	0,063	0,00	0,018	0,01	0,007	0,00	0,000	0,000	0,337
Myrcene	0,862	0,03	0,181	0,04	0,178	0,00	0,043	0,01	0,000	0,00	0,033	0,00	0,000	0,000	0,000
trans-Geraniol	0,000	0,00	0,102	0,01	0,088	0,00	0,019	0,00	0,009	0,00	0,014	0,00	0,000	0,000	0,000
**Esters**	80,934	5,53	42,887	6,54	38,535	5,16	6,528	0,04	3,584	0,04	6,981	0,73	0	0,001	0,001
2-Methylbutyl isobutyrate	1,456	0,08	0,846	0,31	0,749	0,09	0,178	0,03	0,032	0,00	0,090	0,01	0,000	0,010	0,040
Butyl acetate	0,000	0,00	0,004	0,00	0,001	0,00	0,002	0,00	0,000	0,00	0,003	0,00	0,533	0,039	0,000
Butyl caproate	0,000	0,00	0,000	0,00	0,083	0,02	0,000	0,00	0,000	0,00	0,000	0,00	0,000	0,000	0,000
Ethyl acetate	2,874	0,09	2,941	0,50	2,504	0,50	0,236	0,01	0,158	0,01	0,286	0,03	0,000	0,695	0,419
Ethyl butyrate	0,332	0,05	0,344	0,05	0,359	0,07	0,040	0,01	0,039	0,02	0,078	0,02	0,000	0,546	0,923
Ethyl caproate	4,490	0,13	7,516	1,93	5,815	0,10	0,634	0,02	0,453	0,02	0,950	0,11	0,000	0,110	0,070
Ethyl decanoate	21,871	3,07	2,985	0,55	5,076	1,30	1,202	0,07	0,317	0,01	0,766	0,08	0,000	0,000	0,000
Ethyl heptanoate	0,048	0,01	0,286	0,05	0,250	0,04	0,012	0,00	0,005	0,00	0,011	0,00	0,000	0,002	0,002
Ethyl laurate	3,752	0,65	0,382	0,07	0,462	0,10	0,102	0,03	0,056	0,00	0,091	0,02	0,000	0,000	0,000

Ethyl nonanoate	0,560	0,07	0,218	0,08	0,271	0,03	0,036	0,00	0,017	0,01	0,042	0,00	0,000	0,003	0,004
Ethyl octanoate	37,024	2,01	20,107	3,51	17,590	2,46	3,231	0,09	1,987	0,02	3,629	0,36	0,000	0,001	0,001
Ethyl-9-decenoate	0,070	0,01	1,050	0,18	0,505	0,04	0,002	0,00	0,079	0,01	0,100	0,02	0,000	0,000	0,000
Hexyl acetate	0,173	0,01	0,288	0,05	0,295	0,07	0,018	0,00	0,021	0,00	0,042	0,01	0,000	0,063	0,138
Hexyl butyrate	0,000	0,00	0,003	0,00	0,020	0,00	0,001	0,00	0,000	0,00	0,000	0,00	0,000	0,000	0,000
Hexyl caproate	0,000	0,00	0,000	0,00	0,001	0,00	0,002	0,00	0,004	0,00	0,004	0,00	0,001	0,017	0,212
Hexyl n-valerate	0,205	0,03	0,038	0,00	0,076	0,01	0,001	0,00	0,004	0,00	0,020	0,01	0,000	0,000	0,000
Isoamyl acetate	7,557	0,28	5,270	0,87	3,927	0,51	0,730	0,02	0,382	0,05	0,807	0,06	0,000	0,003	0,003
Isoamyl caprote	0,000	0,00	0,000	0,00	0,002	0,00	0,001	0,00	0,001	0,00	0,003	0,00	0,004	0,000	0,551
Isoamyl propionate	0,000	0,00	0,201	0,04	0,170	0,03	0,020	0,01	0,002	0,00	0,007	0,00	0,000	0,001	0,000
Isobutyl isobutyrate	0,521	0,12	0,407	0,09	0,380	0,07	0,079	0,00	0,025	0,00	0,050	0,01	0,533	0,039	0,000
**Other**	2,898	0,36	4,912	0,43	4,692	0,84	0,490	0,11	0,217	0,07	0,552	0,04	0,000	0,033	0,019
Caprylic acid	2,045	0,28	4,019	0,58	4,087	0,79	0,463	0,11	0,208	0,07	0,534	0,04	0,000	0,026	0,018
**Totale**	113,96	7,78	77,6922	12,25	74,0574	10,30	10,716	0,20	6,238	0,26	12,329	1,21	0,000	0,013	0,016

Two-way analysis of variance (ANOVA) ($p \leq 0.05$). T0: beer before maturation; T30: beer after maturation; Beer CONTR: beer with no apple pomace addition; Beer UNST: beer added with unstarted apple pomace; Beer F: beer added with *Lactobacillus rhamnosus* 1473 fermented apple pomace.
